# Implementation strategies preferred by primary care clinicians to facilitate cancer prevention and control activities

**DOI:** 10.1007/s10865-023-00400-2

**Published:** 2023-04-08

**Authors:** Russell E. Glasgow, Michaela Brtnikova, L. Miriam Dickinson, Jennifer K. Carroll, Jamie L. Studts

**Affiliations:** 1 Department of Family Medicine, University of Colorado School of Medicine, 1844 Kona St. Eugene, Aurora, CO OR 97403-2142 USA; 2 Adult and Child Center for Health Outcomes Research and Delivery Science (ACCORDS), University of Colorado School of Medicine and Children’s Hospital Colorado, Aurora, CO USA; 3 Department of Pediatrics, University of Colorado School of Medicine, Aurora, CO USA; 4 American Academy of Family Physicians National Research Network, Leawood, KS USA; 5 Division of Medical Oncology, Department of Medicine, University of Colorado School of Medicine, Aurora, CO USA; 6 University of Colorado Cancer Center, Aurora, CO USA

**Keywords:** Implementation strategies, Context, Cancer prevention and control, Primary prevention, Engagement, Primary care

## Abstract

Key clinical and community members need to be involved in the identification of feasible and impactful implementation strategies for translation of evidence-based interventions into practice. While a wide range of implementation strategies has been developed, there is little research on their applicability for cancer prevention and control (CPC) efforts in primary care. We conducted a survey of primary care physicians to identify implementation strategies they perceive as most feasible and impactful. The survey included both primary prevention behavior change counseling and cancer screening issues. Analyses contrasted ratings of feasibility and impact of nine implementation strategies, and among clinicians in different settings with a focus on comparisons between clinicians in rural vs. non-rural settings. We recruited a convenience sample of 326 respondents from a wide range of practice types from four practice-based research networks in 49 states and including 177 clinicians in rural settings. Ratings of impact were somewhat higher than those for feasibility. Few of the nine implementation strategies were high on both impact and feasibility. Only ‘adapting to my practice’ was rated higher than a 4 (“moderate”) on both impact and feasibility. There were relatively few differences between rural and non-rural clinicians or associated with other clinician or setting characteristics. There is considerable variability in perceived impact and feasibility of implementation strategies for CPC activities among family medicine clinicians. It is important to assess both feasibility and impact of implementation strategies as well as their generalizability across settings. Our results suggest that optimal strategies to implement evidence-based CPC activities will likely need to be adapted for primary care settings. Future research is needed to replicate these findings and identify practical, implementation partner informed implementation strategies.

## Introduction

There is a large gap and time lag between the evidence on effective cancer prevention and control (CPC) strategies and their implementation in diverse clinical settings (Hall et al., 2018; Khan et al., 2021; Neta, 2021; Sauer et al., 2017). This is true for both primary prevention and cancer screenings (Fisher et al., 2011). To design effective, practical programs and strategies to address real world CPC challenges it is important to understand the complex settings in which they are to be implemented (Luig et al., 2018). Implementation challenges are especially acute in rural primary care settings (Saman et al., 2019), which have fewer resources to conduct preventive interventions among patient populations that have many social determinants of health challenges and higher rates of morbidity and mortality (Blake et al., 2017; Charlton et al., 2015).

To design programs that will have wide uptake, be successfully implemented, and sustained, it is important to prioritize engagement of clinical and community partners in design of appropriate interventions (Brownson & Proctor, 2018) and identification of feasible and impactful implementation strategies (Meissner et al., 2020). Although a wide range of implementation strategies have been identified and employed (Powell et al., 2015), there is little research on their applicability for different CPC issues or settings (Rabin & Glasgow, 2015). While there has been considerable recent attention to the issue of engagement, including clinician involvement in design and adaptation of *interventions* in primary care (Luig et al., 2018; Saman et al., 2019), and some on staff in rural settings (Harry et al., 2020; Saman et al., 2019), there has been almost no research on clinicians’ perspectives on different *implementation strategies*.

Obtaining clinician input on implementation strategies that are both feasible and effective constitutes an important opportunity for both advancing implementation science and for providing pragmatic assistance to primary care and other practice settings (Powell et al., 2015, 2017). There is a large number of implementation strategies that differ widely on time and cost, expertise required, etc. (Proctor et al., 2013) We are not aware of comparative data on which strategies are superior or preferred by different types of primary care implementers or practice settings (e.g., rural vs. urban; experienced vs. novice practitioners; high vs. low resource settings). It is likely (Powell et al., 2017) that the optimal and preferred strategies will vary by issue, context, and other factors (Chambers et al., 2013; Waltz et al., 2019). We consider overall helpfulness to be a function of both feasibility and reach, and impact or effectiveness. To achieve improvement in population health, both are needed.

We conducted a primary care clinician survey as part of our NCI funded Implementation Science Center in Cancer Control (ISC3) (Oh et al., 2021). The purpose of the Colorado P50 center grant, *Pragmatic Implementation Science Approaches to Assess and Enhance Value of Cancer Prevention and Control in Rural Primary Care (COISC3),* is to develop, implement, evaluate, and disseminate pragmatic implementation science approaches to cost and value to enhance CPC in rural primary care. We focus especially on tailoring and adapting implementation strategies and intervention approaches to local contexts, populations, settings, and resources (Shelton et al., 2020).

In summary, it will be useful for both research and practice in CPC, and especially research-practice partnerships, to have information about clinician perspectives on which implementation strategies are most feasible and most impactful in general, for different CPC issues, and for particular contexts. To address this issue, we conducted an exploratory study involving a convenience survey of primary care clinicians. The purpose of this report is to (1) identify CPC activities with which clinicians would most like assistance; (2) evaluate perceived impact and feasibility of different implementation strategies to assist with delivery of CPC services; and (3) investigate clinician and practice characteristics potentially related to CPC activities with particular focus on rural vs. non-rural comparisons.

## Methods

This study was reviewed by the Colorado Multiple Institutional Review Board at the University of Colorado, approved as expedited research, and written informed consent was not required. This survey was conducted from September to December 2021.

### Settings and target population

A national sample of primary care clinicians was obtained from the American Academy of Family Physicians (AAFP) combined with clinicians from four practice-based research networks (PBRNs) which are collaborating with our COISC3 Center. Eligible respondents were adult primary care clinicians including doctors of medicine, doctors of osteopathic medicine, nurse practitioners and physicians’ assistants who were providers of record. The vast majority of the practices in these networks were family medicine, which reflected the ‘implementation laboratory partners’ in our NCI funded center. While the study focused on rural primary care, both rural and non-rural clinicians were surveyed to allow comparison.

### Survey design and development

The survey instrument (see Appendix 1) covered the following conceptual areas: perceived need for assistance to improve various CPC services; preferences for assistance with CPC vs. other types of preventive service activities (this issue is the topic of a separate publication); (Brtnikova et al., 2022) perceived feasibility and impact of different implementation strategies; and physician and practice characteristics. The survey underwent internal evaluation and revision by members of the investigative team including cancer researchers (*n* = 5), local primary care clinicians (*n* = 4), and PBRN directors having large numbers of rural practices (*n* = 3). The penultimate version was pilot-tested by a group of 5 cancer researchers and practicing primary care physicians from different regions of the country. More detail on the survey design, development, sample and procedures are available elsewhere (Brtnikova et al., 2022).

#### Needs for assistance with CPC activities

To provide context and specificity for rating the implementation strategies, respondents were asked to select one of seven different CPC activities that they ‘would most like help with implementing’ in their practice. The seven activities included (1) primary prevention behavior change counseling activities (i.e., assessment and counseling on nutrition and diet; physical activity; tobacco use; and HPV vaccine; (2) screening for lung cancer and colorectal cancer; and (3) support for cancer survivorship care.

#### Implementation strategies

To minimize respondent burden, it was necessary to limit the number of implementation strategies rated. Pilot testing indicated that it was feasible to rate only 8–10 strategies. We selected implementation strategies (Powell et al., 2015) from different ERIC conceptual categories (Waltz et al., 2015) that were most applicable to primary care and preventive services. Items were selected from two sources. First, we selected an item from each of five clusters of Expert Recommendations for Implementing Change (ERIC) strategies identified by Waltz et al. (2015) These items were: audit and feedback (from the evaluative and iterative strategies cluster); facilitation (from the interactive assistance cluster); adapt to our practice (from the adapt and tailor to context cluster); training and education for staff (from the train and educate stakeholders cluster); and engage patients (from the engage consumers cluster). Because of our focus on prevention and primary care, we also included items from each of the four categories of implementation strategies identified as used most frequently in the national Evidence Now project that targeted improvement of multiple preventive services in over 200 primary care settings in 12 states (Perry et al., 2019). These implementation strategies were: (1) build a health information technology tool; (2) assess and redesign clinic workflow; (3) refer patients to community resources; and (4) use a quality improvement approach. Pre-testing, cognitive testing, and piloting activities indicated that it was necessary to slightly modify the wording of some items to fit the vocabulary and understanding of practicing primary care clinicians rather than implementation researchers. We intended to have respondents rate implementation strategies for different CPC activities, but pilot testing revealed that respondents were only able to confidently provide answers for activities they desired assistance. Also, time constraints prevented having respondents rate strategies for more than the CPC activity most relevant to that clinician.

#### Ratings of feasibility and impact of implementation strategies

Respondents were asked to provide two ratings for each implementation strategy: perceived feasibility and impact. The rationale was that many evidence-based interventions and strategies identified in efficacy and effectiveness research may not be feasible for many settings or populations (Glasgow & Emmons, 2007). Feasibility was defined for respondents as “how easy it would be to conduct this strategy in your practice”. Impact was defined as “the effect this strategy would have on facilitating consistent delivery in your practice”. Both feasibility and impact were rated on 6-point Likert-type scales. This allowed us to calculate an Overall Fit (helpfulness) index by multiplying impact and feasibility responses for each item, as well as to create a figure that illustrated the combination of feasibility and impact. All implementation strategies rated were presented in a random order across respondents to control for potential order effects.

#### Physician and practice characteristics

Several variables were included for descriptive purposes and as potential moderating factors. Related to our COISC3 Center goals, of greatest a priori interest and a focus of many of our analyses was a classification of rural vs. non-rural clinical settings. Rural status was assessed using zip code of the respondent and the corresponding RUCA codes 4–10 (U.S. Department of Agriculture ERS 2010). Other respondent and practice characteristics included clinician specialty and gender, number of adult patients seen, years since training, size and type of practice (e.g., federally qualified health center; private practice; hospital system), implementation climate (Jacobs et al., 2014), presence of any disease registries or prompting systems, and estimated age group, insurance type, and race/ethnicity characteristics of their patient population.

#### Survey administration

A convenience sample was recruited using Dillman’s Tailored Designed Method, (Dillman & Christian, 2014). All clinicians received an electronic cover letter endorsed by their PBRN or national organization together with a 19-item questionnaire. Those with a known email address received an initial survey using Qualtrics^XM^ and up to two emailed reminders. Due to rules and mandatory practices regarding surveys in different PBRNs, slightly different follow-up procedures were used after the identical initial mail distribution. Those without an email address received a paper survey sent via standard mail. Email non-responders from two PBRNs also received a mailed survey. Each respondent was offered an incentive of $50 using RewardsLink. Due to confidentially concerns we did not have information on nonrespondents to compare characteristics of those who responded vs. did not.

### Statistical analyses

All statistical analyses were performed using SAS software (SAS 9.4, SAS Institute, Cary, NC). Analyses were primarily descriptive and focused on means, standard deviations, and ranges for the implementation strategies. To compare ratings on a) different CPC activities and b) different strategies (with repeated responses for individuals) general linear mixed effects modeling was used for continuous measures with random effect for individual respondent, adjusted for clinician characteristics that impacted results (Ramon et al., 1999). As appropriate, chi-square or ANOVA was used to evaluate potential differences associated with continuous or categorical data on physician and practice characteristics. As this was an exploratory study, we did not conduct formal power analyses, but our resulting sample size of 326 allowed a power of 0.84 to detect an effect size of 0.4, using a two-sided alpha level of 0.01 to adjust for multiple comparisons. (https://www.ai-therapy.com/psychology-statistics/power-calculator).

## Results

### Sample

We received a total of 326 eligible surveys. Respondents practiced in 49 states and included 177 rural and 149 non-rural providers. The overall response rate for completed surveys was 4%, after removing those ineligible (e.g., not providers, not practicing in the U.S., not having adult patients; and incomplete responses). Table 1 summarizes overall clinician and practice characteristics and Table 2 displays characteristics by and differences between rural vs. nonrural providers. As would be expected given the networks which are participating with our center, most respondents were family physicians (91%). There was a wide range of practice types, sizes and number of patients seen. Almost half (47%) of respondents’ patient panels were over 50 years of age; an estimated 32% had Medicare insurance, and 31% had Medicaid insurance (23%) or were uninsured (8%). Clinicians estimated that 66% of their patients were non-Hispanic White, 15% were Latinx, 12% African American and 5% or less were Asian and American Indian/Alaskan Natives.

**Table 1:** Respondent characteristics overall

	Overall (*n* = 326)
Practice location, % (*n*)	
Rural	54% (177)
Non-rural	46% (149)
Type of practice, % (*n*)	
FQHC	16% (49)
Private practice	38% (112)
Hospital/health-system owned	39% (116)
Academic	13% (40)
Other (VA, HMO)	1% (4)
Registry or prompting system for cancer prevention and control services, % (*n*)	
Very robust	31% (92)
For some	46% (139)
No	23% (68)
Degree, % (*n*)	
MD	77% (251)
DO	11% (37)
NP	5% (15)
PA	4% (14)
Other (eliminated from survey)	3% (9)
Specialty, % (*n*)	
Family physician	91% (293)
Internal medicine	6% (19)
Other	4% (12)
Gender, % (I)	
Male	47% (148)
Female	53% (167)
Panel size (patients per week), mean (SD)	73 (103)
Years from finished clinical training, years (SD)	20 (12)
Total number of clinical staff members, mean (SD)	18 (19)
Patient age, mean % (SD)	
Percent < 18 years old	13% (11)
Percent 18–50 years old	39% (15)
Percent > 50 years old	47% (18)
Patients’ insurance types, mean % (SD)	
Percent uninsured	8% (12)
Percent medicaid	23% (18)
Percent medicare	32% (16)
Percent private	36% (21)
Patient’s race and ethnicity, mean % (SD)	
Percent white or caucasian	66% (24)
Percent hispanic or latino	15% (17)
Percent black or African American	13% (16)
Percent Asian	5% (8)
Percent other (American Indian, Alaska native, Native Hawaiian or Pacific Islander)	4% (9)

*FQHC* = Federally qualified health center; *VA* = Veterans affairs, *HMO* = Health maintenance organization; *SD* = Standard deviation; *MD* = Doctor of medicine; *DO* = Doctor of osteopathic medicine; *NP* = Nurse practitioner; *PA* = Physician’ assistant

**Table 2:** Respondent characteristics for rural and nonrural clinicians (*n* = 326)

	Rural *n* = 177	Nonrural *n* = 149	*p* value* (Rural vs. nonrural)
Practice location, % (*n*)	54%	46%	
Type of practice^a^, col %			
FQHC	21%	11%	**0.025**
Private practice	31%	45%	**0.008**
Hospital/health-system owned	45%	32%	**0.019**
Academic	8%	20%	**0.002**
Other (VA, HMO)	4%	0%	0.179
Registry or prompting system for cancer prevention and control services, col %			0.460
Very robust	31%	30%	
For some	44%	50%	
No	25%	20%	
Degree, col %			**0.008**
MD	73%	81%	
DO	10%	13%	
NP	7%	2%	
PA	5%	3%	
Other (eliminated from survey)	5%	0%	
Specialty, col %			
Family physician	91%	91%	0.821
Internal medicine	4%	8%	0.116
Other	5%	1%	**0.039**
Gender, col %			0.577
Male	46%	48%	
Female	54%	52%	
Panel size (patients per week), mean (SD)	67 (45)	79 (142)	0.328
Years from finished clinical training, years (SD)	18 (12)	22 (12)	**0.005**
Total number of clinical staff members, mean (SD)	17 (19)	19 (19)	0.339
Patient age, mean % (SD)			
Percent < 18 years old	13 (10)	14 (12)	0.780
Percent 18–50 years old	37 (16)	42 (15)	**0.018**
Percent > 50 years old	49 (19)	44 (17)	**(0.012)**
Patients’ insurance types, mean % (SD)			
Percent uninsured	8 (11)	8 (13)	0.881
Percent medicaid	25 (18)	22 (18)	0.132
Percent medicare	36 (17)	29 (13)	**0.0002**
Percent private	32 (19)	41 (22)	**0.0004**
Patient’s race and ethnicity, mean % (SD)			
Percent white or Caucasian	72 (22)	60 (24)	** < .0001**
Percent Hispanic or Latino	14 (16)	17 (19)	0.142
Percent black or African American	8 (13)	17 (18)	** < 0.0001**
Percent Asian	4 (8)	6 (7)	**0.014**
Percent other (American Indian, Alaska Native, Native Hawaiian or Pacific Islander)	5 (11)	3 (5)	0.084

*FQHC* = Federally qualified health center; *VA* = Veterans affairs, *HMO* = Health maintenance organization; *SD* = Standard deviation; *MD* = Doctor of medicine; *DO* = Doctor of osteopathic medicine; *NP* = Nurse practitioner; *PA* = Physician’ assistant

^*^Chi-Square for comparison between rural and nonrural (*p* < 0.05 in bold)

^a^Type of practice: percentages within rural and nonrural exceed 100% because respondents could select all that applied

#### Need for assistance across CPC areas

Thirty two percent of clinicians chose nutrition and diet as the CPC area in which they would most like help; 16% chose lung cancer screening; 12% chose each of physical activity, colorectal cancer screening, and cancer survivorship support, 11% chose tobacco use assessment and counseling and 5% HPV discussion. There were no differences on which CPC activity assistance was selected as most desired between clinicians in rural and non-rural settings.

### Ratings of implementation strategies

#### Impact

In general, the various implementation strategies were rated as having moderate impact as summarized in Table 3 (*M* = 3.95 on the 6-point scale). Three of the strategies–‘Engaging patients for tailoring’ (*M* = 4.32), ‘Adapting to our practice’ (*M* = 4.17), and ‘Training and education’ (*M* = 4.12) had more than moderate impact (> than 4). Referring patients to community resources was rated as having little impact, especially among rural clinicians. There was moderate variability on impact ratings within and across the implementation strategies.

**Table 3:** Means and standard deviations on ratings of impact, feasibility and overall fit by rural and nonrural status (*n* = 326)

Implementation strategy	Impact mean (SD)		Feasibility mean (SD)		Fit mean (SD)	
	Rural	Nonrural	Rural	Nonrural	Rural	Nonrural
Assess and redesign clinic workflow	3.73 (1.42)	3.91 (1.22)	3.25 (1.30)	3.42 (1.23)	13.16 (8.64)	14.31 (8.15)
Have a practice facilitator or coach	3.87 (1.46)	4.17 (1.33)	2.94 (1.55)	3.05 (1.53)	12.46 (9.24)	13.65 (9.13)
Refer patients to community resources (e.g., WIC, YMCA, Quitline)	**3.21** (1.33)	**3.62** (1.31)	**3.29** (1.39)	**3.75** (1.35)	**11.50** (7.70)	**14.65** (8.86)
Use a quality improvement approach	3.96 (1.20)	4.06 (1.18)	3.97 (1.32)	3.94 (1.27)	16.62 (8.51)	17.08 (8.98)
Training and education for practice staff	4.13 (1.20)	4.11 (1.21)	3.87 (1.28)	4.05 (1.22)	16.66 (8.44)	17.32 (8.28)
Adapt evidence-based intervention (or guideline) to our practice	4.18 (1.17)	4.16 (1.19)	4.18 (1.16)	4.26 (1.17)	17.95 (7.99)	18.42 (8.29)
Use audit and feedback or some type of periodic data reporting	3.70 (1.22)	3.82 (1.24)	3.65 (1.27)	3.44 (1.18)	14.29 (7.87)	13.99 (7.83)
Engage patients to help create an individually tailored action plan	4.34 (1.21)	4.30 (1.21)	3.77 (1.17)	3.72 (1.21)	16.93 (8.10)	16.79 (8.31)
Build a health information technology tool (e.g., EHR reminder or decision aid)	3.94 (1.39)	3.92 (1.36)	3.33 (1.44)	3.42 (1.38)	13.96 (8.86)	14.18 (8.13)
Overall Mean across all strategies	3.90 (0.93)	4.01 (0.89)	3.58 (0.92)	3.67 (0.88)	14.84 (6.32)	15.60 (6.30)

Means in bold were significantly different between rural and nonrural respondents

After forming subgroups based on the CPC activity chosen, we compared average ratings on impact among categories of (1) primary prevention (nutrition, physical activity, smoking cessation, or HPV counseling), (2) cancer screening (lung, colorectal) and (3) cancer survivorship support. Although average impact ratings were slightly lower among those selecting cancer survivorship (*M* = 3.74 vs. 4.02 for primary prevention activities and 3.90 for cancer screening), these differences were not significant.

There were no differences between rural and non-rural clinicians with the exception that the impact of referral to community resource strategy was rated significantly lower in rural than non-rural practices (*p* < 0.008). Exploratory subgroup analyses evaluating the impact of other practice characteristics indicated that only gender was significant: females rated the implementation strategies as having higher impact (*p* < 0.03) than males. Inclusion of gender in the analysis did not alter results concerning rural-non-rural comparisons.

#### Feasibility

Ratings of feasibility (*M* = 3.62) were lower than those for impact, with the exceptions of making adaptions to one’s practice and referral to community resources (Table 3). The implementation strategy rated as the most feasible was adapting to local context. Practice facilitation was rated as the least feasible strategy, especially for rural clinicians. Once again there was considerable variability across clinicians for the same strategy, suggesting that contextual factors may be important. There were few meaningful differences on feasibility ratings between rural and non-rural clinicians. Rural clinicians did rate the strategy of community referrals as less feasible than non-rural clinicians (*M* of 3.3 vs. 3.8, *p* < 0.004). The only other clinician, practice, and patient population characteristic to moderate results was that clinicians serving a higher percentage of uninsured patients rated implementation strategies as less feasible (*p* < 0.03). Inclusion of this variable in the analysis did not alter results concerning rural-non-rural comparisons.

Although average feasibility ratings were lower among those selecting cancer survivorship as their top area of need (*M* = 3.30 vs. 3.68 for primary prevention activities and 3.66 for cancer screening), these differences were not significant.

#### Overall fit

**Fig. 1:**
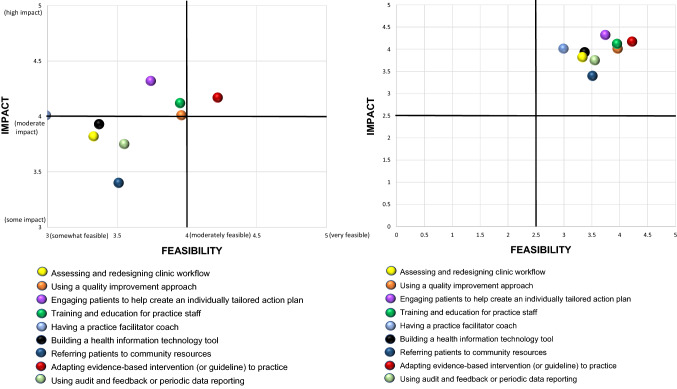
Scatter plot of mean ratings of implementation strategies on impact and feasibility (*n* = 326)

To evaluate the overall helpfulness of implementation strategies, we calculated an overall ‘fit to context’ score by multiplying the feasibility and impact ratings for each strategy as seen in the right-hand columns of Table 3. These scores could range between 1 and 36 and as can be seen, most scores were in the middle to lower range of possible scores: a score of moderate on both feasibility and impact would result in a score of 16. These scores as well as the scatter plot diagram divided into quadrants in Fig. 1 illustrate that only four strategies: adaptations to local practice; training and education; quality improvement; and engaging patients for tailoring received relatively strong fit scores (> 16). The correlation between rated feasibility and impact was 0.68 (*p* < 0.001) indicating that these characteristics while conceptually distinct were far from independent.

Clinician and practice characteristics related to Fit scores were similar to those observed for Impact and Feasibility scores. The only difference between rural and non-rural clinicians was on community referrals with rural clinicians having significantly lower Fit scores than non-rural (*p* < 001). No other clinician or practice characteristics were related to overall Fit scores.

Average FIT scores were marginally lower (*p* < 0.06) for respondents selecting cancer survivorship as their top area of need for assistance (*M* = 13.4) vs. primary prevention activities (*M* = 15.6) or cancer screening (*M* = 15.2) which did not differ.

Figure 1 presents a scatterplot illustrating mean ratings of results for impact and feasibility. As can be seen, only the strategy of adapting evidence-based intervention or guideline to practice had a mean rating falling into the upper right quadrant of having both moderate or greater impact and feasibility. Four strategies- referring patients to community resources; using audit and feedback or periodic reporting; building a health information technology tool; and assessing and redesigning clinic workflow had mean ratings falling into the bottom left quadrant of less than moderate ratings on both impact and feasibility.

## Discussion

This study adds to the literature on clinician perspectives on CPC areas in which clinicians desire assistance. It also advances the implementation science literature on the characteristics and practicality of implementation strategies in the primary care context. In general, the strategies were rated as higher on impact than feasibility. An important recommendation for future research is to consider the combination of both feasibility and impact for selecting implementation strategies. This issue is similar to the broader issue of considering level of adoption and reach of interventions in addition to their clinical effectiveness (Glasgow, 2008; Glasgow & Emmons, 2007; Glasgow et al., 2013) to impact population health.

Few of the nine implementation strategies were high on both impact and feasibility, suggesting that optimal strategies to implement evidence-based CPC activities will likely need to be developed or adapted for primary care settings. Supporting this interpretation, only ‘adapting to my practice’ was rated higher than a 4 (“moderate”) on both impact and feasibility. In contrast, some of the strategies such as practice facilitation were not viewed as feasible, especially in rural settings. Referring patients to community resources was not seen as either feasible or impactful, especially in rural settings, possibly reflecting the relative dearth of CPC resources in these settings.

There was moderate variability on ratings of each implementation strategy. Respondents used the entire 1–6 range in rating the feasibility and impact of all the strategies. With few exceptions this variability was not explained by rural vs. non-rural differences, type of CPC activity being rated, or other clinician, practice, or patient populations factors. This study investigated only a handful of quantitative practice and clinician characteristics. There are multiple possible factors that may have influenced results or obscured differences related to these factors including ease of implementation, availability of resources or other unmeasured influences. Due to survey length restrictions, it was only possible to investigate a few clinician and practice characteristics. More investigation of context—and changing context (Pfadenhauer et al., 2017) is needed. It may also be that ‘micro-tailoring’ is needed: that there is not one category of strategies that is generally more preferred, but that strategies need to be selected in each practice to deal with practice-specific contexts.

This research is an important early step in identifying implementation strategies likely to be most useful for delivering evidence-based CPC services in primary care. Other steps could include (1) evaluating if actual ‘observed feasibility’ (implementation consistency) and impact (effectiveness) are similar to these clinician ratings of projected impact and feasibility (Damschroder et al., 2022; Reilly et al., 2020) and (2) investigating additional ERIC strategies (Powell et al., 2015; Waltz et al., 2015) or those created by practice teams. Implementing and studying CPC activities in primary care is a challenging undertaking, including complexities such as that context, personnel, and competing demands. Implementation strategies often change over time, (Kirk et al., 2020) and strategies are often used in combinations or strategy bundles (Miller et al., 2020). Thus, sequential or contingent selection and tracking of strategies may be important.

This report has several strengths and some limitations. Its strengths include the moderately large sample size and especially the good sized sample of rural primary care clinicians; a variety of practice types, clinician and patient panel characteristics; the random order of presentation of implementation strategies across respondents to control for potential order effects; inclusion of strategies from multiple ERIC categories and those found be applicable in primary care settings; (Perry et al., 2019) and comparison of results for primary prevention, cancer screening and survivorship support activities. We were also able to conduct some subgroup comparisons, especially those relevant to rural-non-rural differences for which there are often insufficient sample sizes to conduct such analyses.

Key limitations include the relatively low survey return rate despite following many best survey research practices recommended by Dillman (Dillman & Christian, 2014) and others (Brtnikova et al., 2018) inclusion of signed, strong letters of support from PBRN leaders and a $50 stipend. This return rate and possibly the observed ratings may be due at least in part to the challenges of coping with COVID-19 and in some cases even the tenuous continued existence of the practices of the clinicians surveyed. In contrast, response rates in surveys to physicians before COVID-19 outbreak were as low as 11% (Cook et al., 2016). Consequently, this is a convenience rather than representative sample; is composed primarily family physicians; and does not represent the perspectives of patients, internal medicine physicians or other practitioners (e.g., PAs and NPs who deliver many services in some rural and low resource practices). Due to the knowledge required to make ratings and survey length restrictions, we only had respondents rate the CPC area in which they most need assistance, rather than have clinicians rate strategies for each CPC activity which would have had methodological advantages.

Although we have hypotheses concerning why the observed pattern of results was obtained, without supporting qualitative data or experimental tests of such interpretations we cannot be confident in these explanations. There was also considerable variability across clinicians, suggesting that there may be other unmeasured contextual factors that influenced our results.

## Conclusions

This study obtained clinician perspectives on the estimated feasibility and impact of different implementation strategies. Impact ratings were moderate and feasibility ratings somewhat lower across a variety of CPC activities, clinicians and types of primary care settings. There were few differences observed between clinicians in rural and non-rural settings on feasibility, impact, or overall ‘fit’ of strategies, despite the well documented challenges of rural CPC (Blake et al., 2017; Charlton et al., 2015). Future research is needed to replicate and expand these findings with different settings and populations, including other types of preventive service activities and implementation strategies.

## Data Availability

The datasets during and/or analyzed during the current study available from the corresponding author on reasonable request.

## Survey on primary care priorities for prevention activities

We would like to understand how you think about and prioritize prevention and screening for different chronic illnesses among your adult patients. This survey should take approximately 10 min. You will be compensated with a $50 gift card, or you may elect to donate the $50 to one of several charities. To be eligible for the incentive, you need to be one of the following: Doctor of Medicine (MD), Doctor of Osteopathic Medicine (DO), Nurse Practitioner (NP), or a Physician's Assistant (PA).

Your responses will be confidential. Results will be reported only in summary fashion. You, and if relevant, your practice-based research network will receive summary results of the survey.

*This survey is being conducted as part of the Pragmatic Implementation Science Approaches to Assess and Enhance Value of Cancer Prevention and Control in Rural Primary Care Project funded by The National Cancer Institute. The research study is based at the University of Colorado | Anschutz Medical Campus. Participation in this survey is completely voluntary. By answering the questions and submitting the questionnaire you are providing consent and permission to use the information shared. If you have questions, you can contact Bryan Ford at bryan.ford@cuanschutz.edu or 303.724.3422. If you have questions about your rights as a participant, you can call the COMIRB (the responsible Institutional Review Board) at 303–724-1055. We believe this research study presents no risk to all participants; if you do not want to respond to any question, simply skip it. There may be risks the researchers have not thought of. This study is not designed to benefit you directly. The data we collect will be used for this study but may also be important for future research. Your data may be used for future research or distributed to other researchers for future study without additional consent if information that identifies you is removed from the data.*

## Code availability

Codes will be shared upon request.
